# Association of Haptoglobin Polymorphism With Microvascular Complications in Type 2 Diabetes Mellitus: A Case-Control Study From a Tertiary Care Center in South India

**DOI:** 10.7759/cureus.104800

**Published:** 2026-03-06

**Authors:** Prabakaran Vaithinathan, Alex Roy X, Sakthivel Vaithiyanathan, C Ganesan, Sridurga Mattyvanan

**Affiliations:** 1 General Medicine, Vinayaka Mission’s Medical College and Hospital, Vinayaka Mission’s Research Foundation (Deemed to be University), Karaikal, IND; 2 Biochemistry, Vinayaka Mission’s Medical College and Hospital, Vinayaka Mission’s Research Foundation (Deemed to be University), Karaikal, IND

**Keywords:** diabetic nephropathy, diabetic neuropathy, diabetic retinopathy, haptoglobin polymorphism, microvascular complications, type 2 diabetes mellitus

## Abstract

Background

Type 2 diabetes mellitus (T2DM) is a major public health problem in India, with microvascular complications contributing substantially to morbidity. Haptoglobin (Hp) polymorphisms, particularly the Hp2-2 genotype, have been associated with increased oxidative stress and vascular dysfunction. This case-control study aimed to evaluate the association between Hp polymorphisms and microvascular complications in T2DM patients attending a tertiary care center in South India.

Methods

A hospital-based case-control study was conducted between July 2023 and December 2024. Eighty-four T2DM patients were enrolled, including 42 cases with documented microvascular complications (nephropathy, retinopathy, and/or neuropathy) and 42 controls without complications. Haptoglobin genotypes (Hp1-1, Hp2-1, Hp2-2) were determined using polymerase chain reaction with sequence-specific primers. Clinical and biochemical parameters, including glycated hemoglobin (HbA1c), lipid profile, and renal function markers, were recorded. Group comparisons were performed using appropriate parametric and non-parametric tests. A p-value <0.05 was considered statistically significant. The study was approved by the Institutional Ethics Committee, and informed consent was obtained from all participants.

Results

The Hp2-2 genotype was significantly more prevalent among cases compared with controls (62.5-83.3% across complication subgroups vs. 26.2% in controls). Significant associations were observed between Hp2-2 and diabetic nephropathy (p=0.001), neuropathy (p=0.003), and retinopathy (p=0.002). Patients with complications demonstrated higher mean HbA1c levels (8.51 ± 2.20% vs. 7.92 ± 1.29%, p=0.038) and a greater prevalence of microalbuminuria (78.6% vs. 11.9%, p<0.001).

Conclusion

The Hp2-2 genotype is strongly associated with the presence of microvascular complications in patients with T2DM. These findings suggest that Hp genotyping may have potential utility in identifying individuals at increased risk for diabetic microvascular disease.

## Introduction

Type 2 diabetes mellitus (T2DM) is a chronic metabolic condition marked by insulin resistance or decreased secretion of insulin and a relative deficiency of insulin, leading to sustained hyperglycemia [1]. India is home to one of the world’s largest populations of individuals with diabetes, with more than 77 million adults affected in 2021 and projections suggesting a rise beyond 134 million by 2045 [2].

Microvascular complications, including diabetic neuropathy, retinopathy, and nephropathy, are frequently observed in patients with prolonged duration of T2DM and contribute significantly to disability, impaired quality of life, and increased healthcare burden [3]. The development of these complications is largely driven by long-standing hyperglycemia, which promotes oxidative stress, inflammatory pathways, and progressive microvascular damage involving neural, retinal, and renal tissues [4].

Haptoglobin (Hp) is an acute-phase glycoprotein encoded by the HP gene located on chromosome 16q22.1 and functions primarily to bind free hemoglobin released during intravascular hemolysis, thereby limiting hemoglobin-mediated oxidative injury [5]. Genetic polymorphism within the HP gene gives rise to three common genotypes-Hp1-1, Hp2-1, and Hp2-2-which differ in structural properties and antioxidant efficiency [6]. Among these variants, the Hp2-2 genotype has been shown to exhibit reduced hemoglobin-scavenging capacity, resulting in heightened oxidative stress and increased susceptibility to vascular injury [7]. Several studies have reported associations between Hp2-2 and an elevated risk of both cardiovascular and microvascular complications in individuals with diabetes [8].

Diabetic neuropathy commonly presents with sensory loss, neuropathic pain, and an increased risk of foot complications, largely attributable to microvascular ischemia and oxidative neuronal injury [9]. Diabetic retinopathy remains a leading cause of vision impairment, progressing from early non-proliferative changes to advanced proliferative disease through chronic retinal hypoxia and pathological neovascularization [10]. Diabetic nephropathy, characterized by persistent albuminuria and declining renal function, represents the most common cause of end-stage renal disease globally [11]. Reduced antioxidant defence associated with the Hp2-2 genotype may exacerbate these pathological processes by intensifying oxidative and inflammatory damage within the microcirculation [12].

In South India, where diabetes prevalence is particularly high, the role of Hp polymorphisms in the development of microvascular complications has not been adequately explored. Regional genetic diversity, dietary factors, and variability in healthcare access may influence the relationship between Hp genotype and complication risk [13]. Although studies from other populations have demonstrated an association between Hp2-2 and adverse diabetic outcomes [8,14], data from this region remain limited.

The present study was therefore undertaken to assess the distribution of Hp genotypes among patients with T2DM with and without microvascular complications attending a tertiary care center in South India and to evaluate their associations with clinical and biochemical parameters, including glycated hemoglobin, lipid profile, and renal function indices. Improved understanding of these relationships may help identify individuals at higher risk for diabetic microvascular disease and inform future preventive strategies.

## Materials and methods

Study setting and participants

This case-control study was conducted at a tertiary care hospital in South India from July 2023 to December 2024. Adult patients aged 18 years and above with a confirmed diagnosis of T2DM were enrolled. Diagnosis was established according to World Health Organisation criteria, including fasting plasma glucose ≥126 mg/dL, 2-hour plasma glucose ≥200 mg/dL, or glycated haemoglobin (HbA1c) ≥6.5%.

Cases (n=42) consisted of patients with at least one documented microvascular complication, namely diabetic nephropathy, retinopathy, and/or neuropathy. Controls (n=42) were patients with T2DM without clinical or laboratory evidence of microvascular complications. Patients with DM-type I, other causes of diabetes mellitus, pregnancy, severe systemic illness, incomplete medical records, or those unwilling to provide informed consent were excluded from the study.

Sample size and sampling

The sample size was calculated for an unmatched case-control design using the standard formula:

N = $frac{\left(Z_{\alpha/2} + Z_{\beta}\right)^2 \left(p_1 q_1 + p_2 q_2\right)}{\left(p_1 - p_2\right)^2}$

where p1 was assumed as 0.50, p2 as 0.20, α as 0.05, and statistical power as 80%.

Based on this calculation, a minimum of 42 participants was required in each group. Eligible participants were selected using systematic random sampling from the hospital diabetes registry.

Clinical and laboratory assessment

Demographic and clinical information were collected using a structured proforma. Biochemical investigations, including glycemic indices, lipid profile, and renal function tests, were performed using an automated chemistry analyser (Erba Chem-8, Transasia Bio-Medicals Ltd., Mumbai). Complete blood counts were analysed using a Sysmex XN-550 haematology analyser (Japan).

Microvascular complications were assessed through fundus examination for diabetic retinopathy, 10-g monofilament testing for diabetic neuropathy, and measurement of urine albumin-to-creatinine ratio, with values exceeding 30 mg/g considered indicative of nephropathy.

Haptoglobin genotyping

Genomic DNA was isolated from EDTA-anticoagulated whole blood samples using the standard phenol-chloroform extraction method. Haptoglobin genotyping was performed by polymerase chain reaction (PCR) employing sequence-specific primers targeting the Hp1 (1500 bp) and Hp2 (3411 bp) alleles.

PCR amplification was conducted in a final reaction volume of 25 μL, with cycling conditions consisting of initial denaturation at 95°C for 5 minutes, followed by 35 cycles of denaturation at 94°C for 30 seconds, annealing at 60°C for 45 seconds, and extension at 72°C for 90 seconds, with a final extension at 72°C for 7 minutes. Amplified products were separated on 1.5% agarose gel electrophoresis and visualised under ultraviolet illumination. For quality control, 10% of samples were randomly selected and re-genotyped.

Ethical considerations

The study protocol was reviewed and approved by the Institutional Ethics Committee of the participating hospital (Approval number VMMC/2023/APR/05). Written informed consent was obtained from all study participants before enrollment. All procedures were conducted in accordance with the principles outlined in the Declaration of Helsinki.

Statistical analysis

Statistical analysis was performed using SPSS software version 25.0 (IBM Corp., Armonk, NY, USA). Data distribution was evaluated using the Shapiro-Wilk test. Continuous variables were compared using Student’s t-test or the Mann-Whitney U test, while categorical variables were analysed using the Chi-square test or Fisher’s exact test, as appropriate. Multivariable logistic regression analysis was planned to adjust for potential confounders, including age, sex, and duration of diabetes. A p-value of less than 0.05 was considered statistically significant. As this was an observational study, formal trial registration was not required.

## Results

Patient characteristics

A total of 84 patients with T2DM were enrolled, including 42 cases with microvascular complications and 42 controls without complications. The mean age of cases was 54.8 ± 10.2 years and of controls 56.2 ± 9.8 years, with no statistically significant difference between the groups (p=0.087, Student’s t-test). Males constituted 52.4% of cases and 50.0% of controls. Table 1 presents the age distribution of the study participants.

**Table 1:** Age Distribution of Cases and Controls.

Age Group (years)	Cases (n=42)	Controls (n=42)
41-50	8 (19.0%)	14 (33.3%)
51-60	20 (47.6%)	10 (23.8%)
61-70	9 (21.4%)	15 (35.7%)
71-80	5 (11.9%)	3 (7.1%)

Duration of diabetes

The duration of diabetes differed significantly between the two groups (p=0.042, Chi-square test). A larger proportion of controls had diabetes for ≤5 years (69.0%), whereas cases demonstrated longer disease duration too, having diabetes for more than 10 years (Table 2). 

**Table 2:** Duration of Diabetes Among Study Groups.

Duration (years)	Cases (n=42)	Controls (n=42)
≤5	18 (42.9%)	29 (69.0%)
6-10	12 (28.6%)	8 (19.0%)
11-15	6 (14.3%)	2 (4.8%)
≥16	6 (14.3%)	3 (7.1%)

Prevalence of microvascular complications

Among cases, diabetic nephropathy was the most common complication (38.1%), followed by neuropathy (33.3%) and retinopathy (28.6%). Combined microvascular involvement was present in over half of the cases, with 9.5% of patients exhibiting all three complications (Table 3).

**Table 3:** Prevalence of Microvascular Complications in Cases.

Complications	Number	Percentage
Diabetic nephropathy	16	38.1%
Diabetic neuropathy	14	33.3%
Diabetic retinopathy	12	28.6%
Nephropathy + Neuropathy	6	14.3%
Nephropathy + Retinopathy	8	19.0%
Neuropathy + Retinopathy	5	11.9%
All three	4	9.5%

Haptoglobin genotype distribution

The Hp2-2 genotype was significantly more prevalent among patients with microvascular complications compared with controls. Across individual and combined complication groups, Hp2-2 ranged from 62.5% to 83.3%, whereas only 26.2% of controls exhibited this genotype. The Hp1-1 genotype was absent in all complication subgroups but present in 14.3% of controls. All genotype-complication associations were statistically significant (p<0.05). All reported p-values were derived from Chi-square or Fisher’s exact tests, as appropriate (Table 4).

**Table 4:** Distribution of Haptoglobin Genotypes. Data is presented as N and %.

Group	Hp1-1	Hp2-1	Hp2-2	X^2 ^value	p-value
Nephropathy (n=16)	0	6 (37.5%)	10 (62.5%)	13.82	0.001
Neuropathy (n=14)	0	5 (35.7%)	9 (64.3%)	11.62	0.003
Retinopathy (n=12)	0	4 (33.3%)	8 (66.7%)	12.43	0.002
DN + DNeu (n=6)	0	1 (16.7%)	5 (83.3%)	11.05	0.004
DN + DRet (n=8)	0	2 (25.0%)	6 (75.0%)	11.61	0.003
DNeu + DRet (n=5)	0	2 (40.0%)	3 (60.0%)	8.85	0.012
All three (n=4)	0	1 (25.0%)	3 (75.0%)	9.66	0.008
Controls (n=42)	6 (14.3%)	25 (59.5%)	11 (26.2%)	—	—

Clinical and laboratory parameters

Patients with microvascular complications exhibited significantly poorer metabolic and renal profiles. Cases had higher mean HbA1c levels (8.51 ± 2.20% vs. 7.92 ± 1.29%, p=0.038), a markedly greater prevalence of microalbuminuria (78.6% vs. 11.9%, p<0.001), and elevated serum creatinine (1.8 ± 0.6 vs. 1.2 ± 0.4 mg/dL, p<0.001). LDL cholesterol was higher and HDL cholesterol lower in cases compared with controls (Table 5).

**Table 5:** Comparison of Clinical and Laboratory Parameters. Data is presented as Mean ± SD.

Parameter	Cases (n=42)	Controls (n=42)	t-value	p-value
HbA1c (%)	8.51 ± 2.20	7.92 ± 1.29	t=2.11	0.038
Microalbuminuria >30 mg/g	33 (78.6%)	5 (11.9%)	X^2^=37.6	<0.001
Creatinine (mg/dL)	1.8 ± 0.6	1.2 ± 0.4	t=5.3	<0.001
LDL (mg/dL)	128.4 ± 28.3	112.6 ± 24.7	t=2.7	0.006
HDL (mg/dL)	36.2 ± 8.1	42.5 ± 9.3	t=3.3	0.002

## Discussion

This study demonstrates a significant association between haptoglobin (Hp) polymorphism and the presence of microvascular complications in patients with type 2 diabetes mellitus (T2DM). The observed prevalence of diabetic nephropathy (38.1%), neuropathy (33.3%), and retinopathy (28.6%) in the present cohort reflects the substantial burden of diabetic microvascular disease in this region and is comparable to findings reported among populations with long-standing diabetes [3].

A principal observation of this study is the markedly higher frequency of the Hp2-2 genotype among patients with microvascular complications compared with controls. Across both individual and combined complication groups, Hp2-2 prevalence ranged from 62.5% to 83.3%, while only 26.2% of controls carried this genotype. These findings are consistent with prior reports linking Hp2-2 with increased vulnerability to diabetic vascular complications, a relationship attributed to impaired hemoglobin scavenging and reduced antioxidant activity [5-8]. The absence of the Hp1-1 genotype among patients with complications, contrasted with its presence in a subset of controls, further supports the proposed protective role of Hp1-1 described in earlier experimental and clinical studies [5].

The association between Hp2-2 and diabetic nephropathy was particularly pronounced in this cohort. Similar observations have been reported by Nakhoul et al., who documented a higher prevalence of nephropathy among Hp2-2 carriers compared with individuals carrying the Hp1-1 genotype [15]. Moczulski et al. also demonstrated a more rapid decline in renal function among Hp2-2 individuals, lending biological plausibility to the present findings [14]. The significantly higher rates of microalbuminuria and elevated serum creatinine levels observed among cases further reinforce the link between Hp2-2 and progressive renal injury.

Significant associations were also identified between the Hp2-2 genotype and both diabetic neuropathy and retinopathy. Oxidative stress and endothelial dysfunction are central to the pathogenesis of these complications [9-12], and the reduced antioxidant efficiency associated with Hp2-2 may intensify microvascular damage within neural and retinal tissues. The high prevalence of Hp2-2 among patients with multiple concurrent complications suggests that this genotype may contribute to cumulative microvascular injury affecting more than one organ system.

Glycemic control appeared to act as an important modifying factor in this study. Patients with microvascular complications exhibited significantly higher HbA1c levels compared with controls, consistent with the established association between chronic hyperglycemia and microvascular damage [1,4]. The coexistence of suboptimal glycemic control and the Hp2-2 genotype may therefore create a metabolic environment characterized by heightened oxidative stress and inflammation, increasing susceptibility to microvascular complications.

In contrast to studies conducted in European and East Asian populations [8,14], the present investigation provides region-specific evidence from South India, where genetic heterogeneity, dietary patterns, and disparities in healthcare access may influence disease expression [13]. These contextual factors highlight the importance of population-specific studies when evaluating genetic markers associated with diabetic complications.

The strengths of this study include its clearly defined case-control design, standardized assessment of microvascular complications, and molecular confirmation of Hp genotypes using PCR-based methods. Nevertheless, several limitations should be acknowledged. The relatively modest sample size may limit generalizability, and the cross-sectional design precludes causal inference. In addition, functional measures of oxidative stress and longitudinal follow-up were not incorporated, which would have strengthened the mechanistic interpretation. Potential confounders such as dietary antioxidant intake and medication adherence were also not evaluated.

From a clinical standpoint, the present findings suggest that Hp genotyping may have value as a risk stratification tool in patients with T2DM. Identification of Hp2-2 carriers could allow earlier surveillance for microvascular complications and support intensified metabolic control. Previous studies have suggested that antioxidant-based interventions may offer greater benefit in Hp2-2 individuals [10], although prospective interventional studies are required before such strategies can be recommended for routine clinical practice.

In summary, this study adds to the growing body of evidence implicating the Hp2-2 genotype as an important genetic contributor to diabetic microvascular disease. Incorporation of genetic markers such as Hp polymorphism into risk assessment frameworks may enhance individualized management strategies for patients with T2DM, particularly in high-burden settings such as South India.

Limitations

The small sample size and single-center, case-control design of this study limit the ability to draw conclusions about causality. Small cell counts were used in subgroup analysis, and because of potential sampling uncertainty, the lack of the Hp1-1 genotype among cases should be regarded with caution. It is impossible to rule out residual confounding from glycemic management and the length of diabetes. More extensive prospective multicenter research is required to confirm these results and elucidate their therapeutic relevance.

## Conclusions

This study demonstrates a significant association between the Hp2-2 genotype and the presence of microvascular complications in these patients. Patients carrying the Hp2-2 genotype exhibited a higher prevalence of nephropathy, neuropathy, and retinopathy, along with poorer glycemic control and adverse renal parameters. These findings support the potential utility of haptoglobin genotyping as a tool for identifying individuals at increased risk of diabetic microvascular disease. While the observational nature of the study limits causal inference, the results provide important regional evidence and a rationale for larger prospective studies to further clarify the role of Hp polymorphism in diabetic complications and its value in personalised risk stratification.
